# Immunomodulatory treatment of interstitial lung disease

**DOI:** 10.1177/17534666221117002

**Published:** 2022-08-08

**Authors:** Laura van den Bosch, Fabrizio Luppi, Giovanni Ferrara, Marco Mura

**Affiliations:** Division of Pulmonary Medicine, University of Alberta, Edmonton, AB, Canada; Department of Medicine and Surgery, University of Milano-Bicocca, Milan, Italy; Respiratory Unit, San Gerardo Hospital, ASST Monza, Monza, Italy; Division of Pulmonary Medicine, University of Alberta, Edmonton, AB, Canada; London Health Sciences Centre, Victoria Hospital, 800 Commissioners Road East, Room E6-203, London, ON N6A 5W9, Canada

**Keywords:** immunomodulatory, immunosuppressive, interstitial lung disease, therapy, treatment

## Abstract

Interstitial lung diseases (ILDs) other than idiopathic pulmonary fibrosis (IPF) have an array of immunomodulatory treatment options compared with IPF, due to their inflammatory component. However, there is a relative paucity of guidance on the management of this heterogeneous group of diseases. In ILDs other than IPF, immunosuppression is the cornerstone of therapy, with varying levels of evidence for different immunomodulatory agents and for each specific ILD. Classification of ILDs is important for guiding treatment decisions. Immunomodulatory agents mainly include corticosteroids, mycophenolate mofetil (MMF), azathioprine, methotrexate, cyclophosphamide and rituximab. In this review, the available evidence for single agents in the most common ILDs is first discussed. We then reviewed practical therapeutic approaches in connective tissue disease–related ILD and interstitial pneumonia with autoimmune features, scleroderma-related ILD, vasculitis and dermatomyositis with hypoxemic respiratory failure, idiopathic non-specific interstitial pneumonia, hypersensitivity pneumonitis sarcoidosis, fibrosing organizing pneumonia and eosinophilic pneumonia. The treatment of acute exacerbations of ILD is also discussed. Therapy augmentation in ILD is dictated by the recognition of progression of disease. Criteria for the evaluation of progression of disease are then discussed. Finally, specific protocol and measures to increase patients’ safety are reviewed as well, including general monitoring and serologic surveillance, *Pneumocystis jirovecii* prophylaxis, patients’ education, genetic testing for azathioprine, MMF serum levels and cyclophosphamide administration protocols. Immunomodulatory therapies are largely successful in the management of ILDs and can be safely managed with the application of specific protocols, precautions and monitoring.

## Introduction

Interstitial lung disease (ILD) encompasses a heterogeneous group of pulmonary diseases characterized by inflammation and fibrosis of the lung parenchyma.^1^ The classification of these ILDs is important for informing treatment decisions. Idiopathic pulmonary fibrosis (IPF) is an idiopathic interstitial pneumonia (IIP) clearly distinguished from other subtypes.^1,2^ IPF is characterized by severe, progressive fibrosis and has poor prognosis, with few, but well-defined, treatment options.^2^ Many of the other ILDs, however, have an inflammatory component in addition to a fibrotic one.

In non-IPF ILDs, the process usually starts with alveolitis, developing when CD4 T cells are activated by antigen-presenting cells. As a result, cytokines are released, and alveolar macrophages, T lymphocytes or neutrophils accumulate in the alveoli and interstitium. Persistent inflammation can result in organization into granuloma and often leads to tissue injury and eventual fibrosis.^3,4^ The inflammatory component allows for an array of therapeutic options, with immunosuppression being the mainstay of therapy, while the fibrotic component may or may not be progressive.^1,5^ As immunomodulatory therapies have increased risk of harm in IPF, particularly increased mortality,^6^ diagnostic accuracy is crucial to determine the best treatment course. For simplicity, from now on, we will refer to any non-IPF fibrotic ILD with a fibrotic component (not necessarily progressive) as ‘fibrosing ILDs’.

In this article, in addition to reviewing the literature regarding immunomodulatory therapies, we discuss the evidence for specific treatment approaches, including precautions and monitoring.

## Immunomodulatory therapies

Currently, immunosuppression is still the mainstay of therapy in ILDs other than IPF. Therapies include corticosteroids, mycophenolate mofetil (MMF), azathioprine (AZA), methotrexate (MTX), cyclophosphamide (CYC) and rituximab (RTX) (Table 1).

**Table 1.** Best published evidence with objective lung function data in ILDs for each drug.

Drug	Condition studied	Efficacy observed^a^	Level of evidence^b^	References
Prednisone	Eosinophilic pneumonia	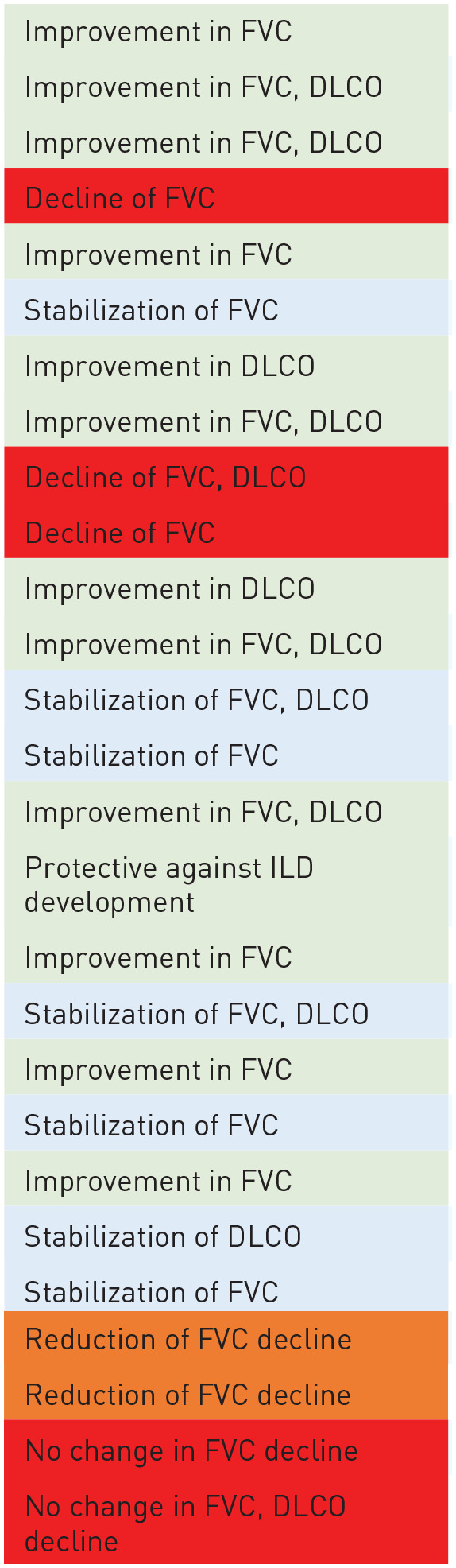	B	Philit *et al.*^7^
	Sarcoidosis		C	Paramothayan and Jones^8^
	HP (non-fibrotic)		C	De Sadeleer *et al.*^9^
	SSc		C	Steen *et al.*^10^
Mycophenolate mofetil	SSc		A	Tashkin *et al.*^11^
	HP		B	Morisset *et al.*^12^
			
	CTD-ILD		C	Fischer *et al.*^13^
	Sarcoidosis		C	Hamzeh *et al.*^14^
Azathioprine	HP		B	Morisset *et al.*^12^
			
	Sarcoidosis		B	Vorselaars *et al.*^15^
	CTD-ILD		C	Boerner *et al.*^16^
	SSc		A	Hoyles *et al.*^17^
Methotrexate	Sarcoidosis		B	Vorselaars *et al.*^15^
	RA-ILD		B	Juge *et al.*^18^
Cyclophosphamide	SSc		A	Tashkin *et al.*^11^
	NSIP		C	Corte *et al.*^19^
Rituximab	SSc		A	Sircar *et al.*^20^
	Sarcoidosis		B	Sweiss *et al.*^21^
	CTD-ILD		B	Duarte *et al.*^22^
			
	RA-ILD		C	Vadillo *et al.*^23^
Nintedanib	SSc		A	Distler *et al.*^24^
	PPF		A	Flaherty *et al.*^25^
Pirfenidone	Unclassifiable ILD		A	Maher *et al.*^26^
	PPF		A	Behr *et al.*^27^

CTD-ILD, connective tissue disease–related interstitial lung disease; DLCO, diffusing capacity of the lungs for carbon monoxide; FVC, forced vital capacity; HP, hypersensitivity pneumonitis; ILD, interstitial lung disease; NSIP, nonspecific interstitial pneumonia; PPF-ILD, progressive pulmonary fibrosis; RA-ILD, rheumatoid arthritis interstitial lung disease; SSc, systemic sclerosis.

a Colour Legend:

Green: improvement

Blue: stabilization

Orange: reduced/slowed rate of decline

Red: decline or no change in amount of decline

b *Level of Evidence Legend*:

A: Randomized clinical trial

B: Multi-centre retrospective study or small (*n* < 20) clinical trial

C: Single-centre retrospective study or systematic review of single-centre studies, and case series

D: Case report/series

### Corticosteroids

Corticosteroids are frequently used as the first-line therapy in ILD for their anti-inflammatory and immunosuppressive effects. Corticosteroids inhibit leukocyte movement and access to inflamed tissues, interfere with leukocyte, fibroblast and endothelial cell function, and suppress humoral factors.^28^ Despite their wide use, there is a surprising lack of high-quality data in ILD.

The efficacy of corticosteroids is dependent, to some extent, on the stage of ILD. Patients with sarcoidosis, cryptogenic organizing pneumonia (COP), acute hypersensitivity pneumonitis (HP) and eosinophilic pneumonia generally respond rapidly and often to a full recovery.^7,8,29^ In a 2018 retrospective study on non-fibrotic HP, corticosteroids increased forced vital capacity (FVC) significantly, but did not have any impact on diffusing lung capacity for carbon monoxide (DLCO) decline.^9^ In fibrosing ILD, although complete reversal is evidently not possible, short-term corticosteroids still have a role in stabilizing rapidly progressive disease.^30^

In connective tissue disease (CTD)-ILD, available data are contrasting^10,31,32^ and corticosteroids are weaned off whenever possible, to avoid long-term side effects. A number of studies have shown benefits, including improved modified Rodnan skin score and improvement or stabilization in pulmonary function tests (PFTs), with steroid combined with other agents in systemic sclerosis–related ILD (SSc-ILD).^33,34^ However, high-dose steroids have been shown to increase the risk of scleroderma renal crisis and are thus often avoided.^35^

### Mycophenolate mofetil

MMF is an immunosuppressant that inhibits inosine monophosphate dehydrogenase and exerts a cytostatic effect on lymphocytes.^36^ MMF is currently the most widely used first-line, steroid-sparing agent in fibrosing ILD as it is generally effective, well tolerated^13,37^ and less toxic than CYC.^11^

In 2016, a randomized trial compared 2 years of MMF therapy with 1 year of oral CYC, followed by 1 year of placebo in SSc-ILD patients. A significant improvement in FVC and Rodney skin score over 2 years was observed with both treatments, with no significant differences between drugs, although MMF was associated with less toxicity.^11^ Both regimens were associated with a significant improvement in the extent of high-resolution computed tomography (HRCT) ILD changes at 2 years^38^ and improvements in health-related quality of life.^39^

MMF was associated with an improvement in or stability of FVC and DLCO in a retrospective study of 125 CTD-ILD patients [including 19 with interstitial pneumonia with autoimmune features (IPAF)] over 2.5 years.^13^ In a study by McCoy *et al.*,^40^ there was a non-significant improvement in FVC and DLCO slope after MMF in IPAF. Similarly, a 2022 study found an association between combination therapy with prednisone and MMF and decreased disease progression.^41^ In myositis-related ILD, a retrospective study found that patients treated with MMF had a significant improvement in FVC and a decrease in mean prednisone dose requirement after 24 months of therapy.^42^

In chronic HP, patients treated with MMF or AZA had a significant improvement in DLCO^12,43^ and reduced prednisone requirements.^43^

Despite its effectiveness in other fibrosing ILDs, MMF has not been shown to be an effective therapy in sarcoidosis. A retrospective study of sarcoidosis patients reported no change in lung function in patients unresponsive to other steroid-sparing agents and treated with MMF for 1 year.^14^

### Azathioprine

AZA is an immunosuppressant agent that inhibits purine synthesis and DNA replication in lymphocytes, and is widely used as the second-line therapy in fibrosing ILD.^44^ Data are unanimously positive, but largely limited to retrospective series.

In SSc-ILD, AZA therapy following intravenous CYC induction has been shown to stabilize or improve lung function in both a 2008 retrospective study and in the randomized Fibrosing Alveolitis in Scleroderma (FAST) trial.^17,45^ In CTD-ILD, AZA has been shown to stabilize or improve lung function during treatment.^16^

In sarcoidosis, AZA is often used as the second-line therapy. A 2013 retrospective study comparing AZA and MTX effect found significant steroid-sparing effect and improvement in FVC and DLCO with both therapies.^15^

In chronic HP, a retrospective study in patients treated with AZA showed significant improvement in FVC after 24 months of treatment.^46^ Other studies showed that chronic HP patients treated with either MMF or AZA had significant improvement in DLCO^12,43^ and reduced prednisone requirements.^43^

### Methotrexate

MTX is a folate analogue that interferes with purine and pyrimidine synthesis and has anti-inflammatory and immunosuppressant effects.^47^ Recent evidence has shown that pulmonary toxicity from MTX is much rarer than previously thought.^48^

In rheumatoid arthritis (RA)-related ILD, MTX does not cause ILD and is actually protective. A 2021 study comparing the use of MTX in patients with RA-ILD to patients with RA without ILD found that ILD detection was significantly delayed in MTX users compared with never-users.^18^ Other studies showed increased survival^49^ and improved lung function^50^ in MTX-treated RA-ILD patients.

In sarcoidosis, MTX is a highly effective second-line therapy after prednisone. In a 2013 retrospective study of 145 patients treated with MTX and 55 with AZA, daily prednisone requirements decreased with both treatments.^15^ In addition, FVC and DLCO increased significantly.^15^ Similar findings were reported by a small randomized trial in patients treated with MTX, compared with placebo.^49^

### Cyclophosphamide

CYC is regarded as the third-line treatment for fibrosing ILD, being more immunosuppressive and toxic, but also as an effective rescue therapy. CYC is a potent alkylating immunosuppressant that is used in numerous hematologic malignancies and autoimmune conditions.^50,51^

The best data were reported in SSc-ILD. The Scleroderma Lung Study I (SLS-I), a randomized, placebo-controlled trial investigating the effect of oral CYC on lung function and symptoms in 145 patients with SSc-ILD across 13 centres, reported a mean absolute difference in FVC of 2.53% between groups (*p* < 0.03) at 12 months, but no significant difference in DLCO. In addition, the CYC arm demonstrated improved dyspnoea and less disability.^52^ Data from the same trial also demonstrated decreased cough frequency with 12 months of CYC, although this was not sustained after discontinuation of therapy.^53^ HRCT changes observed after a year of oral CYC parallel these improvements, with fibrosis being significantly worse in placebo-treated patients.^54,55^ In the SLS II study, CYC and MMF were both effective, but comparatively, MMF was better tolerated.^11^

The multicenter, randomized FAST trial explored the effect of low-dose prednisone and intravenous (iv) CYC for 6 months, followed by maintenance AZA in SSc-ILD. Compared with placebo, predicted FVC in the treatment group improved by 4.2%, but only with a trend towards significance (*p* = 0.08) after 12 months.^17^

Due to potential bladder toxicity, continuing CYC long-term is challenging. In SLS-I, the beneficial effects of 1-year treatment with CYC on lung function and health status dissipated after 18 months, while favourable effects on dyspnea continued through 24 months.^56^ Considering both SLS-I and SLS-II trials, significant improvement in FVC lasted for 12 months, but not beyond that.^57^

In a retrospective study, CYC showed positive results in the treatment of severe progressive nonspecific interstitial pneumonia (NSIP) resistant to other treatments, with stabilization of lung function.^19^ In a 2017 study of iv CYC in patients with steroid-refractory IPAF, an increase in FVC at 6 months was observed (*p* = 0.002).^58^

Notably, CYC is the mainstay of therapy in vasculitis. In antineutrophil cytoplasmic antibody (ANCA)-associated vasculitis, randomized trials have found that prednisone and iv-pulse CYC induce remission as frequently as prednisone and oral CYC.^59^ A 1998 study found similar survival, time of remission and relapse rate between groups,^59^ while a long-term follow-up of patients from the Cyclophosphamide Daily Oral versus Pulsed (CYCLOPS) study found higher risk of relapse with pulse CYC.^60^ Importantly, the total CYC dose is reduced with iv administration.^59^

### Rituximab

RTX is a monoclonal antibody that targets CD20 on B-lymphocytes^61^ and is the object of increasing interest as third- or even second-line option in the therapeutic algorithm of fibrosing ILDs. Although almost all data reported on RTX are positive, there unfortunately is a lack of high-quality trials.

In progressive CTD-ILD, a 2020 retrospective study reported significant improvement in FVC and DLCO after 1 year of treatment with RTX, with sustainable improvement in DLCO remaining at 2 years.^62^ Another retrospective multicentre cohort study on 49 patients with CTD-ILD found stabilization of DLCO and significant improvement of FVC after 1 year of RTX.^22^ In a further retrospective study on CTD-ILD patients, the addition of RTX to MMF reduced daily prednisone requirements, although no significant changes in lung function were seen.^63^

In a 2018 open-label, randomized trial on 60 patients with early SSc-ILD treated with RTX *versus* CYC for 6 months, there was a significant improvement in FVC in the RTX group (from 61% to 68%) and a non-significant decline in the CYC group.^20^ Other studies have had similarly positive findings.^64^ A European prospective, observational, non-randomized study comparing SSc patients treated with RTX to matched, untreated patients, however, did not find any significant difference in FVC or DLCO in the two cohorts over 2 years, but no decline either.^65^

Evidence supporting the use of RTX in RA-ILD has not been as strong. In a retrospective observational study of 44 patients treated with RTX for arthritis, 16% of patients improved and 52% of patients stabilized in terms of FVC, DLCO and radiographic extent on HRCT.^66^ A 2020 study from the Spanish registry found that patients treated with RTX *versus* other therapies had a lower risk of functional decline (decline in FVC ⩾5%).^23^ Another British registry study demonstrated improved survival compared with tumour necrosis factor α (TNFα) inhibitors.^67^

There is a lack of trials exploring the effectiveness of RTX in IPAF; however, in a 2021 case series from two medical centres, 41 of 44 patients with PFTs had improvement or stability in FVC after treatment with RTX.^68^

Idiopathic inflammatory myositis–related ILDs, particularly antisynthetase syndrome-ILD, have shown good response to RTX in observational studies, with stabilization or improvement in radiographic extent^69^ and FVC.^69,70^

RTX has been shown to be an effective rescue therapy in patients with treatment-refractory fibrosing ILD.^71–75^ In a study of 50 severe, progressive ILD patients unresponsive to other immunosuppressants, RTX resulted in a median improvement in FVC of 6.7% (*p* < 0.01) and stabilized DLCO within 6–12 months.^73^ A retrospective, observational study analysed SSc-ILD patients treated with RTX for worsening lung function, despite steroids and immunosuppression with CYC and MMF. Among the 15 patients who completed 2 years of RTX, there was significant improvement in FVC and DLCO.^74^ However, in ‘refractory’ pulmonary sarcoidosis, an open-label, phase I/II trial found inconsistent response to RTX, with only 5 of 10 patients having >5% absolute improvement in FVC.^21^

The Evaluation of Efficacy and Safety of Rituximab with Mycophenolate Mofetil in Patients with Interstitial Lung Diseases (EVER-ILD) trial is a double-blind, placebo-controlled randomized trial currently underway, comparing RTX induction followed by MMF with placebo and MMF in patients with severe and progressive NSIP, refractory to other immunosuppressants.^76^

## Treatment approaches

Aside from cases in which the risk–benefit analysis favours careful observation,^77^ the general approach to immunosuppressive therapy in ILD is based on a dynamic, stepwise process, where treatment is augmented when progression of disease or lack of expected improvement is observed, and is stepped down when lung function has reached a steady plateau.^78^ This approach implies a regular reassessment of treatments and doses in each individual patient (Table 2).

**Table 2.** Suggested approach to treatment by condition.^a^

Condition		Treatments	Approach
CTD-ILD, IPAF		Prednisone	First line^10,31,32,41^
		MMF	First line with prednisone or second line^13,40,41,79^
		AZA	First line with prednisone or second line^16^
		RTX	Third line^22,62,63,68^
		CYC	Third line^58,80^
	RA-ILD	MTX	Second line if required for joint disease^81,82^
		Tocilizumab	Fourth line^83^
SSc-ILD		MMF	First line^11^
		CYC	Second line^11,52^
		RTX	Third line^20,64^
		Tocilizumab	Third line^84^
Vasculitis or Dermatomyositis with hypoxemic respiratory failure		Methylprednisolone pulse	First line^85^
		CYC	First line^59^
		RTX	Second line^75,86^
		AZA	Third line (maintenance only)^87^
		MMF	Third line (maintenance only)^13,88^
NSIP		Prednisone	First line^89^
		MMF	Second line^79^
		AZA	Second line^89^
		CYC	Third line^19,89^
HP		Prednisone	First line^9,29^
	Chronic HP	MMF	Second line^12,43^
		AZA	Second line^12,43,46^
Sarcoidosis		Prednisone	First line^8,89^
		MTX	Second line^15,49^
		AZA	Second line^15^
		RTX	Third line^21^
		Infliximab	Third line^90^
Fibrosing organizing pneumonia		Prednisone	First line^89^
		MMF	Second line^91^
		CYC	Second line^92^
Eosinophilic pneumonia		Prednisone	First line^7^

AZA, azathioprine; CTD-ILD, connective tissue disease related interstitial lung disease; CYC, cyclophosphamide; HP, hypersensitivity pneumonitis; IPAF, interstitial pneumonia with autoimmune features; NSIP, nonspecific interstitial pneumonia; MTX, methotrexate; MMF, mycophenolate mofetil; RA-ILD, rheumatoid arthritis interstitial lung disease; RTX, rituximab; SSc-ILD, systemic sclerosis related interstitial lung disease.

a Nintedanib can be considered for progressive pulmonary fibrosis regardless of the subtype.

A common approach is to start oral prednisone 0.5–1 mg/kg for a limited period of time, to achieve improvement or at least stabilization of disease,^89^ and then to introduce 8–10 weeks later a steroid-sparing agent, to avoid long-term side effects. The steroid is then tapered to a smaller dose and eventually completely stopped, if stabilization of ILD is achieved.

Increasingly, however, steroid-sparing agents such as MMF and AZA are started upfront, especially when the disease at presentation is severe, with supplemental oxygen requirements. In SSc-ILD, where the efficacy of MMF and CYC monotherapy is established,^11,52^ and where corticosteroids may cause a renal crisis,^35^ prednisone may in fact not be used at all. Extrapolating this evidence to other ILDs, MMF or AZA may be used upfront without prednisone, when the absence of rapid progression of disease has been ascertained or when significant contraindications to the use of steroids are present. In the study by Morisset *et al.*,^12^ for example, 77% of patients with HP were treated with either MMF or AZA, without prednisone in advance.

When ILD is clinically significant, with physiologic compromise, an early, complete cessation of immunomodulatory therapy may trigger an acute exacerbation (AE) or rapid progression of disease, with potentially fatal outcome. A cautious, gradual decrease is instead adopted, with the aim of minimizing immunosuppression whenever possible. A complete discontinuance of therapy is possible in sarcoidosis and COP, but not always achieved in other types of ILD.

Since immunosuppressive therapy is often a long-term commitment in ILD, when 2 or more agents are used for a period longer than 2 months, *Pneumocystis jirovecii pneumonia* (PJP) prophylaxis is generally provided. PJP prophylaxis is also usually recommended in the literature with a dose of prednisone ⩾25 mg/day,^93^ although there is no full consensus on this.^94^ The risk of PJP is particularly high in patients receiving an initial dose of ⩾60 mg/day prednisone or equivalent.^93^ In practice, it is therefore generally accepted that high-dose, prolonged courses of prednisone merit PJP prophylaxis, whether as monotherapy or in combination with another agent. In contrast, when a single non-steroid agent is used, PJP prophylaxis is not required, with the notable exception of CYC, which is considered significantly immunosuppressive by itself.^95^ The occurrence of PJP can create major diagnostic difficulties in ILD, as it can be confused with progression of disease or AE. Considering this, and the extremely high morbidity of PJP pneumonia in patients with underlying ILD,^96^ whenever two or more agents are used (either ⩾2 steroid-sparing agents or a steroid-sparing agent in combination with low-dose corticosteroid), PJP prophylaxis is likely indicated.

It is well known that prolonged steroid use accelerates bone loss and increases risk of osteoporosis. Bisphosphonates are therefore recommended in the British Thoracic Society ILD guidelines for ILD patients treated with steroids.^89^ The American College of Rheumatology recommends that all adults taking ⩾2.5 mg/day of prednisone for ⩾3 months optimize their calcium and vitamin D intake. Addition of osteoporosis pharmacotherapy (such as bisphosphonates and denosumab) is based on age and fracture risk.^97^

In aggressive presentations of ILD with severe hypoxemic respiratory failure, presenting with diffuse, bilateral ground glass opacities, such as vasculitis,^98^ dermatomyositis/polymyositis,^99^ or AEs of any fibrosing ILD,^100^ a methylprednisolone iv pulse may stop rapid progression of disease and stabilize the patient. The dose of 10 mg/kg of methylprednisolone iv is usually administered for 3 consecutive days. In rapidly progressive ILD, the institution of very high-dose immunosuppression for a limited period is preferred over a low or average level of therapy with a prolonged treatment course, where adverse events are inevitable.

Surveillance and patient education are both fundamental aspects of the immunomodulatory treatment of ILD to avoid and reduce significant adverse events, as well as increase patient adherence.^78^ While the education of patients and caregivers in clinic is always helpful, it is recommended to also provide written information in lay language about the specific drug(s) used.

Finally, drug-specific protocols of therapy, discussed below, allow further reduction of toxicity.

### MMF serum levels

MMF is a pro-drug of mycophenolic acid (MPA). MMF is rapidly absorbed from the gastrointestinal tract and undergoes extensive pre-systemic de-esterification to become MPA, the active moiety. After an oral dose, MMF in systemic circulation quickly disappears and the plasma concentration of MPA rises rapidly, reaching its maximum concentration within 1 h.^101^ Food intake can delay the rate of MMF absorption, but does not affect the extent of it. Co-administration of antacids or cholestyramine decreases the extent of absorption by approximately 20% and 40%, respectively.^102^

Although not routinely adopted in clinical practice, monitoring of serum levels of MPA can be helpful to ensure therapeutic levels in patients who cannot tolerate the full dose^103^ Unfortunately, there is no published experience on the use of MMF guided by serum levels, but extrapolating the evidence from transplant experience, a level of 1.0–3.5 µg/ml should be targeted.^104^ This approach may potentially allow a reduction in the dose of MMF with improved tolerability, while still achieving a therapeutic level of MPA.

### Genetic testing for AZA

Thiopurine methyltransferase (TPMT) genetic profiling can be used to identify intermediate and slow metabolizers of AZA who are at higher risk of developing bone marrow suppression.^105^ In addition, HLA-DQA1-HLA-DRB genetic profiling can predict the risk of pancreatitis with AZA.^106^ A recent study demonstrated that genetic testing was associated with a significantly reduced incidence of major adverse events and a lower rate of AZA discontinuation, but the total number of adverse events did not change, as available genetic testing does not predict the risk of liver dysfunction or other side effects.^107^ While the cost-effectiveness of systematic genetic testing for AZA has not yet been demonstrated, it is very likely to increase patient safety.

### CYC treatment protocol to reduce toxicity

CYC is the most potent immunosuppressive drug in the pulmonologist’s armamentarium for ILD. The British Thoracic Society recommended the use of iv rather than oral CYC,^89^ given preferable side effect profile.^107^ The rate of leukopenia, severe infections, and gonadal toxicity were reduced in the iv administration route, compared with oral, without differences in patient outcomes.^59^ The recommended iv dose is 500–750 mg/m^2^ monthly,^78^ but frequency can be increased in severe cases with hypoxemic respiratory failure. However, the total dose should not exceed 20 g, as the risk of bladder cancer increases above that level.^108^ It is unusual to exceed 12 g in a treatment course of ILD.

To reduce the risk of hemorrhagic cystitis and bladder cancer, the administration of 250–500 ml of normal saline before and after infusion and good hydration for the following 72 h is recommended.^109^ The concomitant administration of ondansetron reduces the frequency of emesis.^110^

The white blood cell nadir usually occurs 10–14 days after an iv pulse, and bi-weekly surveillance is strongly recommended. These precautions, together with dose adjustments dictated by regular surveillance, should ensure a safe administration of CYC in most cases.

## Recognizing progression of disease

Therapy augmentation in ILD is undoubtedly dictated by the recognition of progression of disease. However, there is currently no consensus as to how disease progression should be defined in ILD patients.^24,25,111,112^ A number of end-points have been proposed in clinical trials exploring fibrosing ILDs.^5^ In IPF and other ILDs, most studies have defined disease progression as a decline in FVC, measured as the change from baseline or as a categorical change (typically ⩾10% predicted).^24,25,111,112^ A decline in FVC is a well-defined predictor of mortality in IPF.^113,114^ Nevertheless, a recent study showed remarkable heterogeneity in FVC trajectories, depending on the ILD subtype.^115^ Patient-reported outcomes (PRO), imaging features, acute worsening events, mortality, exercise capacity and quality of life measures are often used as secondary end-points.^5^

In daily clinical practice, progression of ILD is highlighted by the integration of multiple domains, including deterioration in lung function tests, worsening of fibrosis on chest HRCT, worsening of symptoms and exercise capacity. Measurement of FVC and DLCO is considered the best tool in monitoring disease progression. However, the main limitations are represented by test variability and confounding pulmonary comorbidities, such as emphysema or pulmonary hypertension. The recent 2022 American Thoracic Society guideline proposes a definition of progressive pulmonary fibrosis (PPF) as ⩾2/3 of (1) worsening respiratory symptoms, (2) physiologic (absolute fall ⩾5% in FVC and ⩾10% in DLCO within 1 year) and (3) radiographic evidence of progression.^116^

Respiratory symptoms are meaningful in detecting disease progression. Although there are no data on fibrosing ILD, chronic cough in IPF is not only often refractory but is also considered an independent predictor of disease progression.^117^ Similarly, changes in dyspnea score, for example, have been demonstrated to be independently predictive of survival in ILD patients.^118^

PRO and experiences are key to understanding needs and facilitating patient-centred care. Symptoms should be measured across the disease course. In fact, in IPF, PRO measures are considered secondary outcomes in clinical trials.^119^

Reduced exercise capacity is an essential characteristic of progressive fibrosing ILDs, and a decline in 6-min walk distance (6MWD), at least in IPF, is a strong, independent predictor of mortality.^120^ 6MWD can be affected by numerous factors, including age, body size, comorbidities and the use of supplemental oxygen during the test, and these issues need to be considered in result interpretation of both individual and serial tests.^121^

HRCT has a role in staging and quantifying the extent of diffuse lung diseases. However, there currently is a need to create a reproducible HRCT staging system for the evaluation of clinically significant changes. Many studies recognize the extent of fibrosis as a strong predictor of outcome in patients with IPF.^122,123^ However, studies using a visual, semiquantitative score of parenchymal abnormalities to predict the mortality rate are considered poorly reproducible.^124^ Computer-based quantification of disease on CT has been used in a variety of ILDs and have significantly improved human-based CT evaluation.^125–129^ Quantitative CT also has several limitations, mainly related to the fact that it is heavily influenced by CT dose, slice thickness and reconstruction kernel.^126^

When progression of fibrosing ILD occurs, a role for anti-fibrotic therapy may be considered.^24–27,130^ Although this is not the object of this review, given the recent published evidence, we included anti-fibrotic agents in the suggested approaches to therapy (Tables 1 and 2). Combination therapy of MMF or other immunomodulatory agents with either nintedanib or pirfenidone is considered tolerable and safe. The decision as to whether the best management for a patient with progressive phenotypes of ILD is to intensify immunosuppression, introduce second-line therapy with anti-fibrotic therapy, or combine these two approaches is challenging and will require future studies specifically designed to address combination therapy and with well-defined criteria for truly progressive ILD.^131^

## Conclusion

Immunomodulatory therapy is largely successful in the treatment of ILD and can be safely managed with the application of specific protocols, precautions, monitoring and patient education. This is reflected by consistently better outcomes reported for fibrosing ILD other than IPF compared with IPF, despite a remarkable scarcity of clinical trials on immunosuppressive agents. There is currently a key need to clarify the optimal timing and sequence of treatments.

## Declarations

**Ethics approval and consent to participate:** This is a review manuscript and no ethics approval was required.

**Consent for publication:** All authors have read and approved the submitted manuscript. No other consent for publication was required.

**Availability of data and material:** This is a review paper.
